# Efficacy of surgery versus definitive chemoradiotherapy following neoadjuvant chemotherapy in T4 esophageal squamous cell carcinoma: a retrospective cohort analysis

**DOI:** 10.3389/fonc.2026.1736386

**Published:** 2026-03-20

**Authors:** Shiwang Wen, Hesong Wang, Xiaohan Zhao, Jingyuan Wen, Xinyi Liu, Ke Yan, Shuguang Li, Chunyang Song, Wenbin Shen

**Affiliations:** 1 Department of Thoracic Surgery, Fourth Hospital of Hebei Medical University, Shijiazhuang, Hebei, China; 2 Department of Radiation Oncology, Fourth Hospital of Hebei Medical University, Shijiazhuang, Hebei, China

**Keywords:** adjuvant chemoradiotherapy, clinical T4N0-3M0 stage, definitive chemoradiotherapy, esophageal squamous cell carcinoma, neoadjuvant chemotherapy, prognosis, surgery

## Abstract

**Background:**

T4 esophageal squamous cell carcinoma (ESCC) is associated with dismal prognosis, and the optimal treatment remains uncertain. This study aimed to identify the clinical and pathological characteristics of T4 ESCC patients in China and compare the efficacy, adverse events, and treatment failure patterns of two treatment strategies: neoadjuvant chemotherapy followed by surgery and adjuvant chemoradiotherapy (nCT+S) or neoadjuvant chemotherapy followed by definitive chemoradiotherapy (nCT+dCRT).

**Methods:**

We retrospectively analyzed the clinical records of 1242 cT4N0-3M0 ESCC patients from January 2015 to December 2020. After performing propensity score matching (PSM), a total of 195 patients were included in the analysis, comprising 73 patients in the nCT+S group and 122 in the nCT+dCRT group. To address potential selection bias related to resectability, we further performed a subgroup analysis limited to MDT-confirmed resectable T4 ESCC patients after nCT.Endpoints included overall survival (OS), progression-free survival (PFS), and related clinical outcomes.

**Results:**

The median follow-up time was 77.0 months (95% CI: 68.1-85.9). For the entire post-propensity score matching (PSM) cohort, the 1-, 3-, and 5-year overall survival (OS) rates were 65.6%, 27.6%, and 17.4%, respectively, with a median OS of 18.0 months (95% CI:14.9-21.1); the corresponding 1-, 3-, and 5-year progression-free survival (PFS) rates were 49.7%, 22.6%, and 14.5%, with a median PFS of 12.0 months (95% CI:8.8-15.2).Multivariate Cox regression analysis confirmed that treatment modality (nCT+S vs nCT+dCRT) and N stage were independent prognostic factors for both OS (HR = 1.867, 95% CI:1.341-2.598;HR=1.595, 95% CI:1.286-1.977, both P<0.001) and PFS (HR = 1.576, 95%CI:1.146-2.167; HR = 1.741, 95%CI:1.286-1.977, both P<0.01).After Bonferroni correction for multiple testing (corrected α=0.0025), subgroup analysis demonstrated that patients with ECOG performance status 0-1, cT4a stage, cN0 stage, and mid-esophageal tumors derived significant OS benefits from nCT+S (all P<0.0025);for PFS, male patients, those aged >64 years, with tumor length >5.0 cm, cT4a stage, cN0 stage, and mid-esophageal tumors obtained significant benefits from nCT+S (all P<0.0025).In the subgroup restricted to MDT-assessed resectable T4 lesions after nCT, nCT+S still provided significantly superior OS and PFS versus nCT+dCRT (both P<0.0025 after Bonferroni correction).Notably, the incidence of severe treatment-related complications was significantly lower in the nCT+S group (4.1%, 3/73) than in the nCT+dCRT group (18.9%, 23/122; χ²=8.591, P = 0.003).Additionally, the rate of isolated local or regional lymph node recurrence was significantly higher in the nCT+dCRT group (χ²=7.348, P = 0.007), though the overall treatment failure rate showed no statistical difference between the two groups.

**Conclusion:**

In nCT-responsive, MDT-evaluated resectable or downstaged resectable cT4N0-3M0 ESCC, nCT+S achieves longer OS and PFS than nCT+dCRT, with more survival benefits in ECOG 0-1, cT4a, cN0 or mid-esophageal tumor subgroups; it also lowers severe complications and isolated local/regional recurrence risk.These results were validated in a resectable-only subgroup, minimizing selection bias related to tumor resectability.Large multicenter randomized trials are required to validate these findings and define optimal therapy for T4 ESCC due to single-center retrospective limitations.

## Introduction

Esophageal cancer ranks among the most prevalent malignant tumors worldwide, with markedly high incidence rates across Asian countries. China, in particular, accounts for more than half of the global cases of esophageal squamous cell carcinoma (ESCC) (1, 2). Due to its insidious onset in the early stages and the exclusion of invasive diagnostics such as esophagogastroduodenoscopy from routine health screenings, frequently culminates in diagnoses at advanced stage (3). In certain instance, patients are diagnosed with stage T4 cancer, distinguished by the invasion of esophageal tumor to adjacent organs. There is considerable clinical debate regarding the optimal treatment approach for T4 esophageal cancer patients (4, 5). Currently, the standard treatment for patients with locally advanced resectable esophageal cancer involves neoadjuvant chemoradiotherapy followed by surgical resection (6), while some cancer centers, including ours, prefer neoadjuvant chemotherapy (nCT) as a treatment option.

Furthermore, in existing clinical trials, the proportion of T4 ESCC patients is relatively low, with their survival outcomes yet to be dissected in isolation (7, 8). Consequently, there is no consensus on whether T4 ESCC patients should undergo surgical resection or definitive chemoradiotherapy (dCRT) following neoadjuvant treatment.

A meticulous review of the available literature revealed a lack of long-term outcome studies comparing surgery and definitive chemoradiotherapy in patients who respond to neoadjuvant chemotherapy. Moreover, most studies do not separately analyze patients who became resectable after nCT, which may introduce selection bias if surgical patients are inherently more treatable. To address this gap, we analyzed data from a single-center study and further performed a subgroup analysis limited to patients with MDT-confirmed resectable T4 disease after nCT, to provide an alternative treatment approach for T4 ESCC patients who are either unable or unwilling to undergo neoadjuvant chemoradiotherapy.

## Methods

### Study design and patients

This was a retrospective cohort analysis including patients diagnosed with esophageal squamous cell carcinoma (ESCC), confirmed by pre-treatment esophageal endoscopic ultrasonography (EUS) pathology at the Fourth Hospital of Hebei Medical University. Key inclusion criteria included classification as cT4N0-3M0 according to the 8th edition of the esophageal cancer TNM staging system of the Union for International Cancer Control (UICC) and the American Joint Committee on Cancer (AJCC): the diagnosis of cT4a stage strictly followed the above staging system, cT4a was defined as esophageal tumor invading adjacent structures including pleura, diaphragm, azygos vein, pericardium, or lung parenchyma (without invasion of vital organs such as aorta, trachea, or main bronchus); tumor invasion into lung parenchyma was classified as cT4a, while invasion into main bronchus (≥segmental level) or trachea was classified as cT4b, preoperative staging was comprehensively determined by enhanced CT, esophageal EUS, and MDT discussion, patients with bronchus or aorta invasion (cT4b) were mostly included in the nCT+dCRT group due to high surgical risk, individual resectable cases received surgery but all achieved R1 resection;cT4b patients with significant downstaging and MDT-confirmed resectability after nCT were also eligible for surgery.All enrolled patients received at least two cycles of nCT;After receiving neoadjuvant chemotherapy, the patients were evaluated according to the response evaluation criteria in solid tumors (RECIST) version 1.1 as having effective therapeutic effect.All enrolled cT4 ESCC patients were confirmed to have tumor invasion of adjacent organs via preoperative esophageal endoscopic ultrasonography (EUS), enhanced chest/abdominal computed tomography (CT), and positron emission tomography-computed tomography (PET-CT) if necessary, the involved organs included pleura, diaphragm, azygos vein, pericardium, lung, and tracheobronchial tree;The diagnosis of clinically positive lymph node metastasis (cN+) was comprehensively determined by enhanced neck/chest/upper abdominal CT, neck ultrasound, and esophageal EUS, not solely based on size; the criteria included: ① short-axis diameter ≥10 mm, or 5-10 mm with irregular shape, unclear boundary, heterogeneous enhancement; ② abnormal enlargement or clustering of lymph nodes in esophageal drainage areas; ③ hypoechoic, round lymph nodes with loss of hilum structure on EUS, suspected cases were confirmed by PET-CT or MDT discussion.

Exclusion criteria encompassed the presence of other malignant tumors, prior anti-tumor treatments, non-squamous cell carcinoma histology, pre-existing distant organ metastasis, incomplete follow-up information or follow-up time less than 3 months, lack of improvement in esophageal tumor lesions or metastatic lymph nodes after nCT and no prior postoperative adjuvant radiotherapy (PORT).

This study was approved by the Ethics Committee of the Fourth Hospital of Hebei Medical University (approval number: 2025KS008), and informed consents were obtained from all participants prior to treatment.

### Treatments

Patients underwent two distinct treatment regimens: neoadjuvant chemotherapy followed by surgical resection and adjuvant chemoradiotherapy (nCT+S) or neoadjuvant chemotherapy followed by dCRT (nCT+dCRT).After 2-4 cycles of nCT, all patients were evaluated for efficacy (RECIST 1.1, cCR/PR as effective) and resectability by multidisciplinary team (MDT) composed of radiation oncologists, thoracic surgeons, medical oncologists, radiologists, and pathologists. Grouping principles: ① nCT+S group: patients with effective nCT response, resectable tumor (R0/R1 resection feasible), good physical status (ECOG 0-2) and willingness to receive surgical treatment, cT4b patients with significant downstaging and no vital organ invasion were also included if resectable; ② nCT+dCRT group: patients with effective nCT response but unresectable tumo, poor physical status unable to tolerate surgery, or patients who refused surgical treatment.

The chemotherapy regimens primarily consisted of paclitaxel combined with platinum (TP) or 5-fluorouracil combined with platinum (FP). In the TP regimen, paclitaxel (135–175 mg/m²) was administered intravenously on Day 1, with cisplatin (75 mg/m²) given over 1-3 days. In the FP regimen, 5-fluorouracil (500 mg/m²) was administered from Day 1 to Day 5 with cisplatin (75 mg/m²) over 1-5 days. Both regimens were repeated every 21 or 28 days. For nCT, patients received 2-4 cycles, whereas those undergoing dCRT received 4-6 cycles, with a median of 5 cycles. For postoperative adjuvant chemotherapy, patients received 3-6 cycles, with a median of 4 cycles. Patients who received radical chemoradiotherapy received 1-2 cycles of chemotherapy simultaneously with radiotherapy, and received 0-2 cycles of chemotherapy after radiotherapy; Patients who underwent surgery received 1-4 cycles of chemotherapy after surgery.

As for radiotherapy, dCRT regimen included Simultaneous-Integrated Boost Intensity-Modulated Radiation Therapy (SIB-IMRT) with the prescribed dose of 54 Gy/30 fractions to the Planning Target Volume (PTV) and PTV-nd, and 60 Gy/30 fractions to the GTV and GTV-nd, using conventional fractionation.The definitions of radiotherapy target volumes were as follows: GTV (Gross Tumor Volume) referred to the gross volume of the primary esophageal tumor confirmed by preoperative esophageal endoscopy, enhanced CT, and esophageal EUS, including the visible primary tumor lesion on imaging, delineated based on the maximum diameter and scope of the tumor shown by enhanced CT combined with endoscopic tumor location; GTV-nd (Gross Tumor Volume of Nodal Disease) referred to the gross volume of clinically positive lymph nodes confirmed by preoperative enhanced CT, neck ultrasound, esophageal EUS, and PET-CT (if applicable), including all lymph nodes meeting cN+ diagnostic criteria, covering cervical, mediastinal, gastric cardia, and celiac axis lymph node drainage areas with suspected metastasis. The dose of PORT depended on surgical margin status: patients with negative margins received IMRT, with a prescribed dose of 50.4 Gy/28 fractions to the PTV; patients with positive margins received SIB-IMRT with 50.4 Gy/28 fractions to the PTV and 56 Gy/28 fractions to the PTV-p, using conventional fractionation.

Surgical procedures were decided by a multidisciplinary team(MDT) and involved resection of both the primary tumor and metastatic lymph nodes, with an approach tailored to tumor location and patient characteristics.For upper thoracic lesions, a right thoracotomy (Ivor Lewis or McKeown) with extended lymph node dissection was applied; for middle and lower thoracic lesions, a left thoracotomy with extended lymph node dissection was performed.For middle thoracic lesions with suspected right recurrent laryngeal nerve lymph node metastasis (confirmed by preoperative neck ultrasound and enhanced CT), the surgical approach was adjusted to Ivor Lewis or McKeown right thoracotomy to achieve complete lymph node dissection; left thoracotomy could not realize complete dissection of right recurrent laryngeal nerve lymph nodes, thus close follow-up for cervical lymph node recurrence was conducted for these patients, and timely salvage treatment was given if recurrence occurred. The interval between nCT and surgery was 2-6 weeks, with a median of 4 weeks.After esophagectomy, all patients received esophageal-gastric anastomosis for digestive tract reconstruction. Reconstruction routes were selected according to surgical approaches:For McKeown esophagectomy (right thoracotomy with cervical anastomosis), the retrosternal route was used in 89.2% of cases, and the posterior mediastinal route was applied only in individual patients with a narrow retrosternal space.For Ivor Lewis esophagectomy (right thoracotomy with intrathoracic anastomosis), the posterior mediastinal route was uniformly used in all cases.For left thoracotomy, the posterior mediastinal route was adopted in all cases, which was consistent with the original anatomical position and conducive to postoperative gastrointestinal function recovery.Preoperative nutritional support for both groups was standardized based on PG-SGA score: ① patients with PG-SGA score ≤3:oral nutritional supplementation combined with routine diet; ② patients with PG-SGA score 4-8:enteral nutrition support for 2-4 weeks before treatment combined with oral supplementation; ③patients with PG-SGA score ≥9:combined enteral and parenteral nutrition support for 4 weeks before treatment until nutritional status improved (PG-SGA score ≤5).There was no statistically significant difference in preoperative nutritional status and support methods between the two groups (P>0.05).

### Endpoints and assessments

The endpoints of the study were overall survival (OS) and progression-free survival (PFS). OS was defined as the time from the initiation of neoadjuvant chemotherapy to death from any cause, while PFS was defined as the time from initiation of nCT to disease progression, recurrence, or death from any cause.OS and PFS were calculated from the initiation of nCT to ensure consistent baseline and comparability between the two groups;this method reflects the overall benefit of the complete treatment strategy (nCT+S vs nCT+dCRT) and avoids bias caused by different intervals between nCT and subsequent treatment (2-6 weeks for nCT+S group, 1-2 weeks for nCT+dCRT group).

Efficacy assessments were conducted after 2–4 cycles of nCT, using enhanced chest and abdominal Computed Tomography (CT), neck ultrasound, esophageal EUS, whole-body bone scintigraphy, and esophagography (Positron Emission Tomography (PET)-CT was used if necessary). Clinical Complete Response (cCR) was defined as complete disappearance of the tumor, while Partial Response (PR) was defined as a reduction in the longest tumor diameter of at least 30%. Lymph node response was evaluated according to Response Evaluation Criteria in Solid Tumors 1.1 (RECST 1.1) criteria. Safety evaluation included clinical symptoms, hematological tests, and imaging studies such as neck ultrasound and chest/upper abdominal CT. Adverse events (AEs) were graded according to the Common Toxicity Criteria (CTC) 3.0. As for follow-up, in the first two years after treatment, follow-up occurred every 3-6 months; in the next three years, every 6-12 months; and thereafter, annually. Informed consent was written. Our study adhered to the Declaration of Helsinki by stating this in the manuscript.

### Statistical analysis

Statistical analyses were conducted using SPSS 26.0 (IBM Corporation, Armonk, New York, USA). Categorical data were presented as N (%), and group comparisons were performed using the χ² test or Fisher’s exact test, as appropriate. The median follow-up time was estimated using the reverse Kaplan-Meier method. The Kaplan-Meier method was employed to calculate patient survival rates, and differences in survival curves between groups were compared using the log-rank test. Multivariable analysis of independent factors influencing overall survival (OS) and progression-free survival (PFS) was performed using the Cox proportional hazards regression model. To adjust for differences in clinical characteristics between the nCT+S and nCT+dCRT groups, to mitigate selection bias in retrospective data and maximize the large-sample advantage, we conducted 1:N propensity score matching (PSM) via nearest neighbor matching with a caliper of 0.2.After PSM, the standardized mean differences(SMD) of all baseline variables were reduced to less than 0.2, indicating that the baseline imbalance between the nCT+S and nCT+dCRT groups was effectively corrected, and the baseline characteristics were well balanced.A significance threshold of *P* < 0.05 was considered statistically significant. To further address potential bias related to resectability, a prespecified subgroup analysis was performed limited to patients with MDT-confirmed resectable T4 disease after nCT.For subgroup risk analyses involving multiple independent tests, the Bonferroni correction method was applied to adjust the significance threshold to avoid false positive rate inflation: a total of 20 independent tests (10 for OS and 10 for PFS) were included, with the original α=0.05 corrected to α=0.0025. R statistical software (R: A Language and Environment for Statistical Computing. R Foundation for Statistical Computing, version 3.3.2, Vienna, Austria.ISBN 3-900051-07-0, URL http://www.R-project.org/, accessed on 08 November 2022) was used to perform the statistical analyses.

## Results

### Patient characteristics

**Figure 1:**
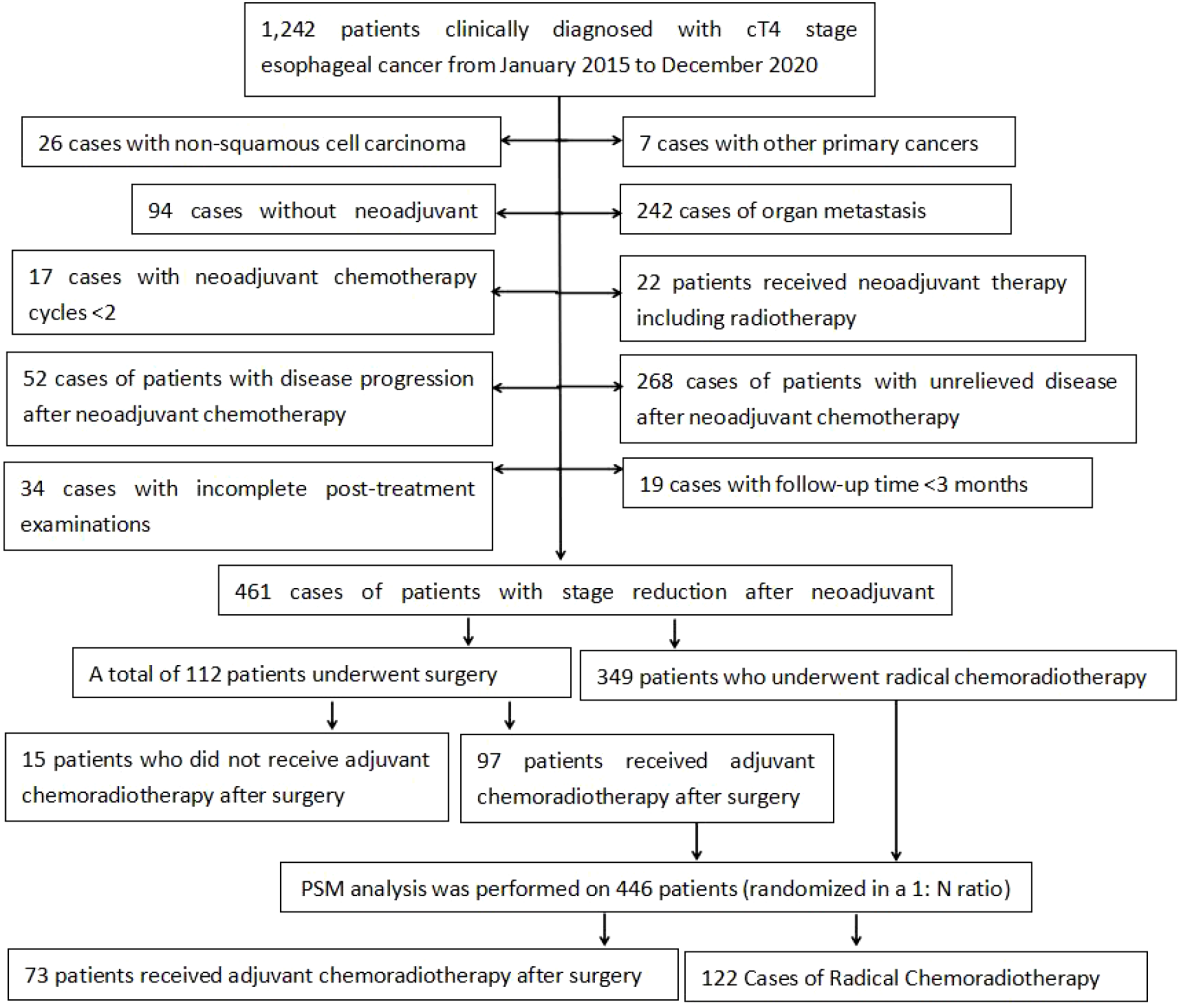
Patient characteristics.

We retrospectively analyzed the medical records of 1, 242 patients with cT4N0-3M0 stage esophageal cancer who were treated at the Fourth Hospital of Hebei Medical University between January 2015 to December 2020.After applying the inclusion and exclusion criteria, a total of 446 patients were included in the final cohort (**Figure 1**). Among the enrolled patients, there were 314 males and 132 females. The age range was 42 to 79 years, with a median age of 64 years. The ECOG performance status scores were distributed as follows: 103 patients scored 0, 123 scored 1, and 220 scored 2. Barium esophagography revealed that the length of the esophageal lesions ranged from 2.0 to 13.0 cm, with a median of 7.3 cm. There were 296 patients with cT4a stage and 150 patients with cT4b stage. In terms of N stage, 185 patients were N0, 198 were N1, and 63 were N2, and no patients had N3 stage.

### Efficacy and prognostic factors

The median follow-up time was 76.5 months (95% CI: 69.8-83.2). The OS rates for the entire cohort at 1, 3, and 5 years were 65.9%, 25.7%, and 14.9%, respectively, with a median OS time of 18.2 months (95% CI: 16.3-20.1). As presented in **Table 1**, univariate analysis identified treatment modality, lesion length, and N stage as significant factors influencing OS (χ² values were 17.740, 4.461, and 17.853, with *P*-values < 0.001, 0.035, and < 0.001, respectively). Multivariate analysis further confirmed that treatment modality and N stage were independent factors influencing OS, with hazard ratios (HR) of 1.687 (95% CI: 1.290-2.206) and 1.325 (95% CI: 1.148-1.529), respectively.

**Table 1:** Analysis of factors affecting OS in 446 patients before PSM.

Indicator	N	Univariate analysis						Multivariate analysis		
		1year(%)	3year(%)	5year(%)	Median time (Month)	χ²	*P*	*P*	HR	95%CI
Treatment						17.740	<0.001	<0.001	1.687	1.290~2.206
nCT+S	97	77.3	37.1	28.9	27.0					
nCT+dCRT	349	62.7	22.5	10.2	17.0					
Gender						0.611	0.435			
Male	314	63.6	25.5	13.7	18.4					
Female	132	71.2	26.5	18.2	18.0					
Age (years)						0.365	0.546			
≤64	230	62.1	24.9	16.1	18.0					
>64	216	69.9	26.7	13.5	18.2					
ECOG						0.004	0.952			
0-1	226	69.4	25.8	13.7	18.8					
2	220	62.3	25.7	16.1	17.1					
Length						4.461	0.035	0.895	1.019	0.773~1.343
≤5.0cm	85	72.8	34.2	19.5	25.0					
>5.0cm	361	64.3	23.8	13.8	17.5					
Location						3.729	0.155			
Upper	97	60.8	22.9	7.6	15.5					
Middle	242	67.3	24.3	16.6	18.8					
Lower	107	67.3	31.3	17.8	18.2					
cTStage						2.488	0.115			
cT4a	296	70.2	27.9	15.3	19.8					
cT4b	150	57.3	21.5	14.1	14.1					
cNStage						17.853	<0.001	<0.001	1.325	1.148~1.529
cN0	185	73.5	37.1	20.7	23.0					
cN1	198	63.1	17.7	10.9	15.5					
cN2	63	52.4	14.4	9.0	15.6					

(*P* <0.05).

The 1-year, 3-year, and 5-year progression-free survival (PFS) rates for the entire cohort were 50.6%, 20.7%, and 12.4%, respectively, with a median PFS of 12.3 months (95% CI: 10.6–14.0). As shown in **Table 2**, univariate analysis results revealed a significant correlation between PFS and treatment modality or N stage (χ² = 9.547, 28.248, with *P* = 0.002 and <0.001). The multivariate analysis also indicated that these two factors were independent factors influencing PFS, with HRs of 1.432 (95% CI: 1.121–1.830) and 1.428 (95% CI: 1.243–1.640), respectively.

**Table 2:** Analysis of factors affecting PFS in 446 patients before PSM.

Indicator	N	Univariate analysis						Multivariate analysis		
		1year(%)	3year(%)	5year(%)	Median time (Month)	χ²	*P*	*P*	HR	95%CI
Treatment						9.547	0.002	0.004	1.432	1.121~1.830
nCT+S	97	64.9	29.9	22.7	18.2					
nCT+dCRT	349	46.7	18.0	8.9	11.0					
Gender						1.096	0.295			
Male	314	49.3	20.7	11.3	12.0					
Female	132	53.8	20.9	15.5	13.0					
Age (years)						0.019	0.889			
≤64	230	49.1	18.7	13.6	12.0					
>64	216	52.3	22.9	10.7	12.7					
ECOG						0.878	0.349			
0-1	226	53.5	21.1	12.2	13.3					
2	220	47.7	20.3	12.5	10.9					
Length						3.540	0.060			
≤5.0cm	85	61.0	25.8	16.0	18.2					
>5.0cm	361	48.2	19.6	11.6	11.6					
Location						1.688	0.430			
Upper	97	45.4	16.3	7.5	11.0					
Middle	242	52.0	20.0	13.9	12.6					
Lower	107	52.3	25.8	13.6	13.0					
cTStage						2.696	0.101			
cT4a	296	55.4	22.0	12.9	13.9					
cT4b	150	42.7	18.1	11.1	8.3					
cNStage						28.248	<0.001	<0.001	1.428	1.243~1.640
cN0	185	58.9	32.9	17.7	18.4					
cN1	198	46.9	12.4	9.2	11.0					
cN2	63	38.1	8.4	6.7	8.0					

(*P* <0.05).

### Patient characteristics post-PSM

As shown in **Table 3**, before PSM, significant differences between the two groups of patients in terms of gender, age, lesion length, lesion location, and cT stage (χ² = 7.250, 7.568, 32.520, 45.132, 18.332, *P* = 0.007, 0.006, <0.001, <0.001, <0.001). After PSM, all baseline characteristics were well balanced, including cT4a/cT4b distribution (SMD = 0.147) and resectability-related factors.Notably, 11 cT4b patients (15.1%) in the post-PSM nCT+S group were included due to MDT-confirmed resectability after nCT, ensuring the group was not limited to cT4a only.

**Table 3:** Comparison of general clinical data composition between two treatment modalities before and after PSM.

Indicator	N	Before PSM[N(%)]		χ²	*P*	SMD	N	After PSM [N(%)]		χ²	*P*	SMD
		nCT+S	nCT+dCRT					nCT+S	nCT+dCRT			
Gender				7.250	0.007	0.293				0.560	0.454	0.101
Male	314	79(81.4)	235(67.3)				152	59(80.8)	93(76.2)			
Female	132	18(18.6)	114(32.7)				43	14(19.2)	29(23.8)			
Age (years)				7.568	0.006	0.339				0.018	0.894	0.018
≤64	230	62(63.9)	168(48.1)				111	42(57.5)	69(56.6)			
>64	216	35(36.1)	181(51.9)				84	31(42.5)	53(43.4)			
ECOG				3.246	0.072	0.209				0.058	0.809	0.037
0-1	226	57(58.8)	169(48.4)				117	43(58.9)	74(60.7)			
2	220	40(41.2)	180(51.6)				78	30(41.1)	48(39.3)			
Length				32.520	<0.001	0.454				3.153	0.076	0.163
≤5.0cm	85	38(39.2)	47(13.5)				60	28(38.4)	32(26.2)			
>5.0cm	361	59(60.8)	302(86.5)				135	45(61.6)	90(73.8)			
Location				45.132	<0.001	0.493				1.707	0.426	0.133
Upper	97	5(5.2)	92(26.4)				18	5(6.8)	13(10.7)			
Middle	242	46(47.4)	196(56.2)				112	46(63.0)	66(54.1)			
Lower	107	46(47.4)	61(17.5)				65	22(30.1)	43(35.2)			
cTStage				18.332	<0.001	0.500				1.158	0.282	0.147
cT4a	296	82(84.5)	214(61.3)				158	62(84.9)	96(78.7)			
cT4b	150	15(15.3)	135(38.7)				37	11(15.1)	26(21.3)			
cNStage				0.383	0.826	0.006				0.470	0.791	0.069
cN0	185	40(41.2)	145(41.5)				76	30(41.1)	46(37.7)			
cN1	198	45(46.4)	153(43.8)				91	34(46.6)	57(46.7)			
cN2	63	12(12.4)	51(14.6)				29	9(12.3)	20(15.6)			
nCT Response				0.042	0.837	0.021				0.031	0.859	0.042
cCR	53	12(12.4)	41(11.7)				17	9(12.3)	8(10.9)			
PR	393	85(87.6)	308(88.3)				129	64(87.7)	65(89.1)			

### Efficacy and prognostic factors post-PSM

**Figure 2:**
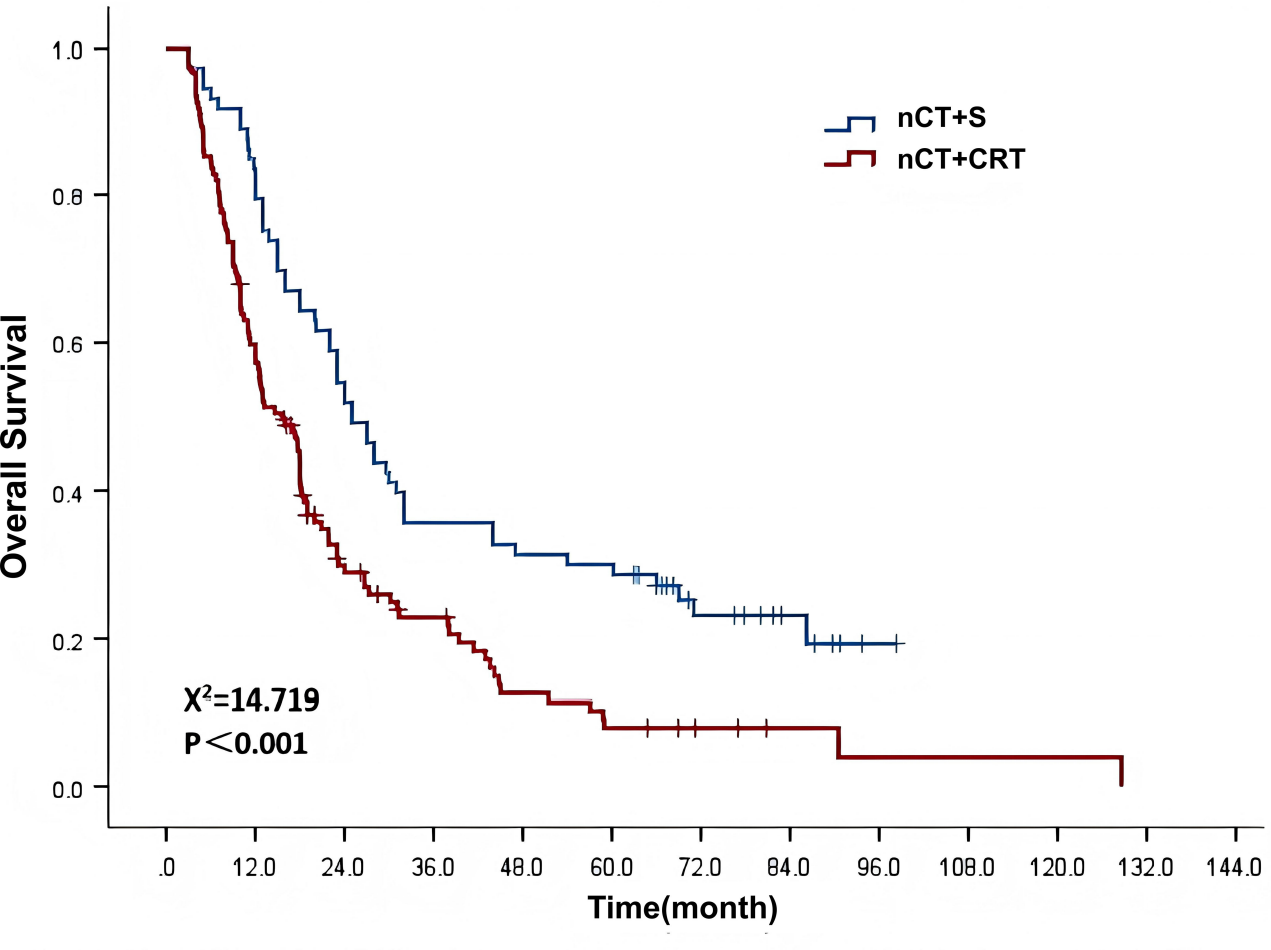
Survival curves showing the impact of different treatment modalities on patient OS.

Among 195 esophageal squamous cell carcinoma patients included in the post-PSM analysis, and in the post-PSM nCT+S group (73 cases), 62 achieved R0 resection (84.9%), 11 achieved R1 resection (15.1%), and no R2 resection cases.The median follow-up duration was 77.0 months (95% CI: 68.1–85.9). The 1-year, 3-year, and 5-year overall survival (OS) rates were 65.6%, 27.6%, and 17.4%, respectively, with a median OS time of 18.0 months (95% CI: 14.9–21.1) (**Figure 2**). As illustrated in **Table 4**, the univariate analysis results indicated that treatment modality and N stage were significant factors influencing OS (χ² = 14.719, 321.308, *P* values < 0.001). The multivariate analysis also showed that these two factors were independent factors influencing OS, with HRs of 1.867 (95% CI: 1.341–2.598) and 1.595 (95% CI: 1.286–1.977), respectively.

**Table 4:** Analysis of factors affecting OS in 195 patients after PSM.

Indicator	N	Univariate analysis						Multivariate analysis		
		1year(%)	3year(%)	5year(%)	Median time (Month)	χ²	*P*	*P*	HR	95%CI
Treatment						14.719	<0.001	<0.001	1.867	1.341~2.598
nCT+S	73	79.5	35.6	30.1	25.0					
nCT+dCRT	122	57.2	22.9	8.0	15.6					
Gender						0.008	0.930			
Male	152	64.4	28.5	15.8	19.0					
Female	43	69.8	24.3	21.8	18.0					
Age (years)						0.813	0.367			
≤64	111	62.9	27.9	20.1	18.0					
>64	84	69.0	27.1	14.2	18.0					
ECOG						1.638	0.201			
0-1	117	67.4	23.7	13.8	18.0					
2	78	62.8	33.6	22.9	23.0					
Length						1.786	0.181			
≤5.0cm	60	69.7	30.0	19.4	21.8					
>5.0cm	135	63.7	26.6	16.5	18.0					
Location						1.868	0.430			
Upper	18	50.0	26.7	6.7	12.0					
Middle	112	66.9	27.1	19.8	21.8					
Lower	65	67.7	28.8	16.2	15.6					
cTStage						0.001	0.969			
cT4a	158	68.3	26.6	15.8	20.0					
cT4b	37	54.1	32.2	25.1	12.8					
cNStage						21.308	<0.001	<0.001	1.595	1.286~1.977
cN0	76	78.9	45.2	29.7	30.0					
cN1	91	62.5	14.6	8.8	17.4					
cN2	28	39.3	17.9	8.9	12.0					

In the post-PSM nCT+S group, Median OS of R0 resection patients was 28.6 months (95%CI:24.3-32.9), significantly longer than 12.3 months (95%CI:8.1-16.5) of R1 resection patients (χ²=19.264, P<0.001);Median PFS of R0 resection patients was 20.1 months (95%CI:16.7-23.5), significantly longer than 8.7 months (95%CI:5.2-12.2) of R1 resection patients (χ²=16.837, P<0.001).

To address reviewer concern about selection bias from resectability, we performed an additional analysis restricted to 158 post-PSM patients with MDT-confirmed resectable T4 disease (cT4a and downstaged cT4b) after nCT.In this resectable-only subgroup:nCT+S (n=62) showed significantly longer OS than nCT+dCRT (n=96) (P<0.001);nCT+S showed significantly longer PFS (P<0.0025 after Bonferroni correction);Severe complication rate remained significantly lower in nCT+S (3.2% vs 17.7%, P = 0.007);Isolated locoregional recurrence was significantly lower in nCT+S (16.1% vs 38.5%, P = 0.009).These results confirm that the survival benefit of nCT+S is not caused by preferential selection of more favorable resectable cases, but represents a genuine treatment advantage.

**Figure 3:**
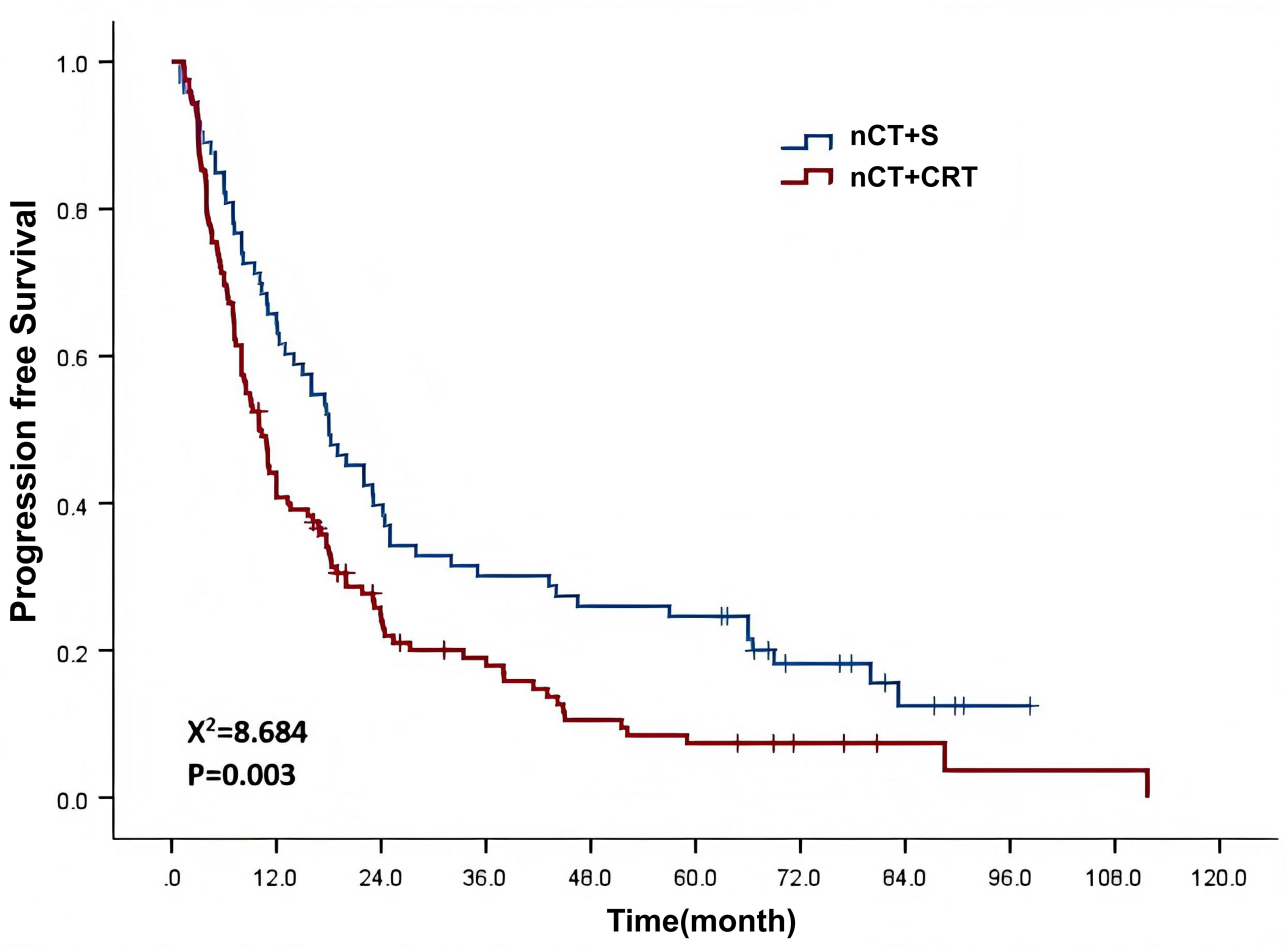
Survival curves showing the impact of different treatment modalities on patient PFS.

The 1-year, 3-year, and 5-year progression-free survival (PFS) rates were 49.7%, 22.6%, and 14.5%, respectively, and the median PFS was 12.0 months (95% CI: 8.8–15.2) (**Figure 3**). As suggested in **Table 5**, univariate analysis results showed that treatment modality and N stage were significant factors influencing PFS (X² = 8.684, 30.804, P = 0.003, <0.001). The multivariate analysis also indicated that these two factors were independent factors influencing PFS, with HRs of 1.576 (95% CI: 1.146–2.167) and 1.741 (95% CI: 1.412–2.147), respectively.

**Table 5:** Analysis of factors affecting PFS in 195 patients after PSM.

Indicator	N	Univariate analysis						Multivariate analysis		
		1year(%)	3year(%)	5year(%)	Median time (Month)	χ²	*P*	*P*	HR	95%CI
Treatment						8.684	0.003	0.005	1.576	1.146~2.167
nCT+S	73	64.4	30.1	24.7	18.0					
nCT+dCRT	122	40.8	17.9	7.4	10.0					
Gender						0.005	0.943			
Male	152	50.5	23.0	13.0	12.1					
Female	43	46.5	20.9	18.6	10.3					
Age (years)						0.251	0.616			
≤64	111	49.4	21.6	17.3	12.0					
>64	84	50.0	23.7	11.2	12.0					
ECOG						0.015	0.902			
0-1	117	51.1	21.4	12.2	12.3					
2	78	47.4	24.6	17.8	11.0					
Length						1.122	0.289			
≤5.0cm	60	56.3	23.3	14.3	16.0					
>5.0cm	135	46.7	22.4	14.7	11.2					
Location						0.957	0.620			
Upper	18	44.4	11.1	5.6	11.0					
Middle	112	52.5	23.5	16.4	15.0					
Lower	65	46.2	24.3	13.9	10.9					
cTStage						0.428	0.513			
cT4a	158	53.1	22.3	14.4	13.6					
cT4b	37	35.1	24.0	15.0	8.0					
cNStage						30.804	<0.001	<0.001	1.741	1.412~2.147
cN0	76	64.5	41.3	26.2	23.0					
cN1	91	44.9	9.6	6.4	10.9					
cN2	28	25.0	10.7	7.1	7.0					

### Subgroup analysis of patients benefiting from different treatment modalities

**Figure 4:**
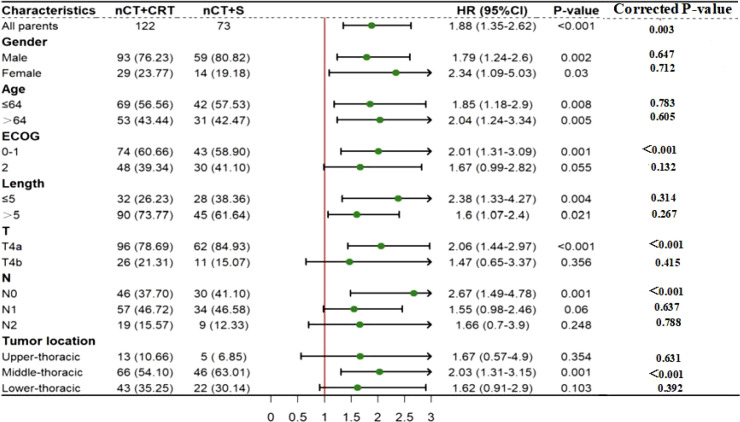
Forest plot of subgroup analysis for OS comparing nCT+S vs nCT+dCRT (after Bonferroni correction, corrected α=0.0025). HR>1 indicates better OS benefit in the nCT+S group; *P<0.0025 (statistically significant after correction).

After Bonferroni correction for multiple testing, as shown in **Figure 4**, patients with better ECOG score (0-1), cT4a stage, cN0 stage, and mid-esophageal tumors demonstrated a more pronounced OS advantage with nCT+S (all P <0.001, <0.0025); no significant OS benefit of nCT+S was observed in subgroups of gender, age, and tumor length (corrected P >0.0025).

**Figure 5:**
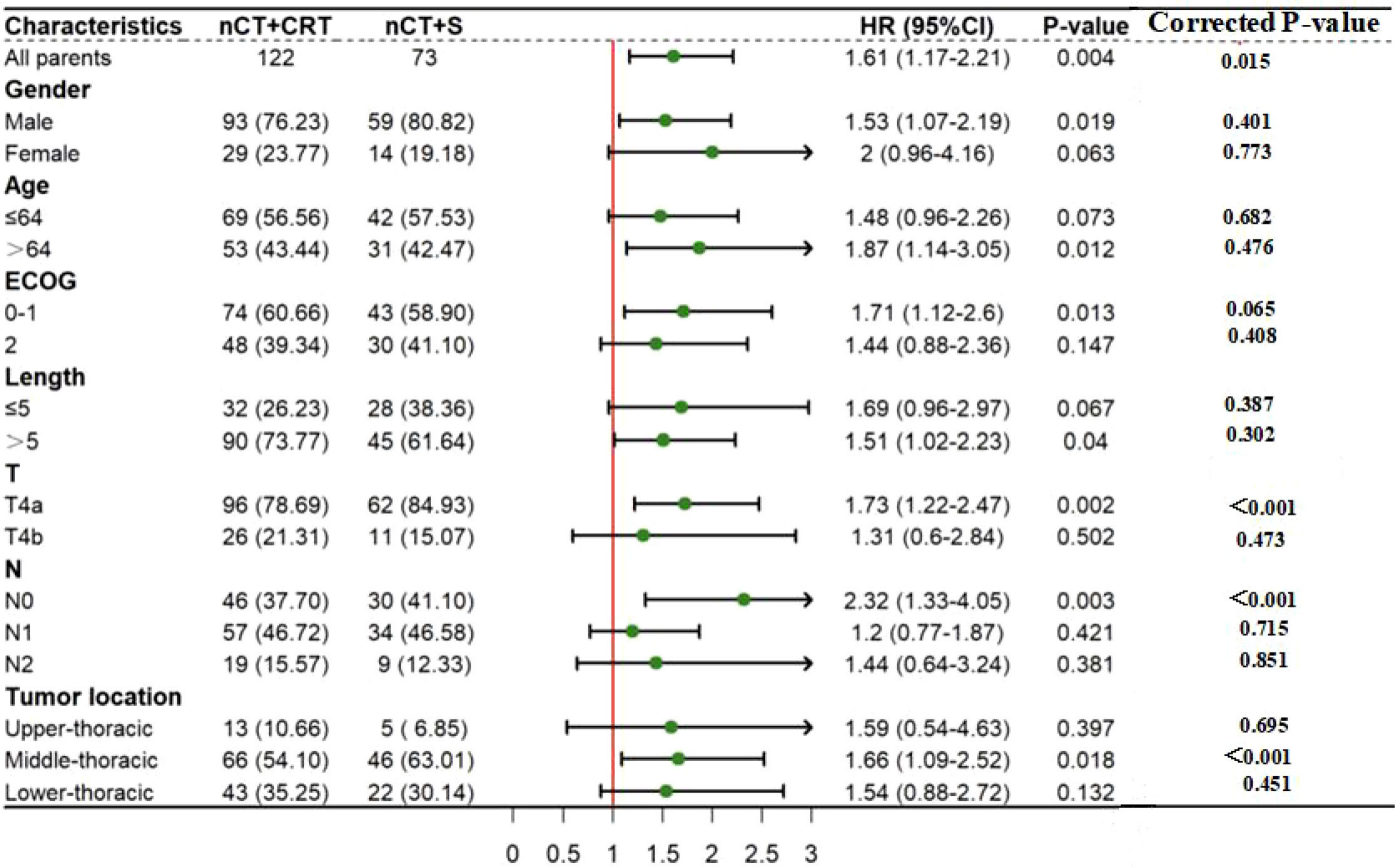
Forest plot of subgroup analysis for PFS comparing nCT+S vs nCT+dCRT (after Bonferroni correction, corrected α=0.0025). HR>1 indicates better PFS benefit in the nCT+S group; *P<0.0025 (statistically significant after correction).

Based on the same Bonferroni correction standard, as suggested in **Figure 5**, patients with cT4a stage, cN0 stage, and mid-esophageal cancer benefited from nCT+S in terms of PFS (all P < 0.001, < 0.0025); subgroups including male patients, those aged >64 years, and with lesion length >5.0 cm showed no significant PFS benefit from nCT+S (corrected P > 0.0025).

### Analysis of treatment-related severe complications and treatment failure patterns post-PSM

In the nCT+S group, severe complications were relatively infrequent, 1 patient (1.4%) experiencing an anastomotic leak and 2 patients (2.7%) had hematemesis. In the nCT+dCRT group, 14 patients (11.5%) had an esophageal fistula and 9 patients (7.4%) had hematemesis. The overall incidence of severe complications in the nCT+S and nCT+dCRT groups was 4.1% (3/73) and 18.9% (23/122), respectively, with a statistically significant difference (χ² = 8.591, *P* = 0.003).

Regarding treatment failure patterns, recurrence at the primary tumor bed and/or regional lymph nodes occurred in 15 patients (20.5%)in the nCT+S group and 48 patients (39.3%) in the nCT+dCRT group; distant organ or lymph node metastasis was observed in 10 patients (13.7%) and 17 patients (13.9%), respectively, while both emerged in 17 patients (23.3%) and 11 patients (9.0%), respectively. The overall incidence of treatment failure in the nCT+S and nCT+dCRT groups were comparable between the two groups, with 57.5% (42/73) in the nCT+S group and 62.3% (76/122) in the nCT+CRT group, showing no statistically significant difference (χ² = 0.433, P = 0.510). However, the incidence of isolated local recurrence at the tumor bed and/or regional lymph nodes was significantly higher in the nCT+CRT group (χ² = 7.348, *P* = 0.007).

## Discussion

This study compared the efficacy, adverse events and patterns of treatment failure in two groups of T4N0-3M0 ESCC patients: those receiving neoadjuvant chemotherapy (nCT) followed by surgery and adjuvant chemoradiotherapy (nCT+S group) versus those undergoing synchronous chemoradiotherapy (nCT+dCRT group). Our findings indicated that the nCT+S group significantly outperformed the nCT+dCRT group in terms of both OS and PFS, with a lower N stage and the nCT+S treatment being independent predictive factors of better prognosis.

Previous studies have also compared different treatment modalities for ESCC (9), yet studies specifically focusing on T4 stage ESCC patients remains relatively limited. Some investigations have reported results aligned with ours, indicating the beneficial impact of neoadjuvant therapy combined with surgery in improving survival rates for ESCC patients (10, 11). However, discrepancies exist among studies, which may be due to difference in patient selection, treatment protocols, and research methodologies. In many cases, broader-stage populations were analyzed without isolating T4 stage ESCC, and difference in neoadjuvant chemotherapy or chemoradiotherapy protocols may have affected the comparability and accuracy of the outcomes.

Despite regional and institutional differences in the adoption of neoadjuvant treatments for esophageal cancer, numerous studies on neoadjuvant treatment, whether reported or ongoing, are available. The OEO2 study in the UK (12) confirmed that nCT improves OS in esophageal cancer patients (the 5-year OS rate for the nCT+S group was 26.4%, compared to 14.3% in the surgery-only group, *P* < 0.001). Similarly, the MAGIC study in the UK (13) found that preoperative and postoperative chemotherapy prolonged OS (the 5-year OS rate for the preoperative and postoperative chemotherapy group was 36%, significantly better than the 23% in the surgery-only group, *P* = 0.009) and PFS (HR = 0.66, 95%CI: 0.53~0.81, *P* < 0.001) in esophageal cancer patients. The JCOG 9907 study in Japan (14) further revealed that preoperative chemotherapy was more effective than postoperative chemotherapy, with a 5-year OS rate of 55% versus 43% (HR = 0.73, 95% CI: 0.54–0.99, *P* = 0.04). Although these studies primarily included T2-3 stage esophageal cancer patients, our study extended the evidence by showing that even among more advanced T4 stage patients, nCT+S significantly improved both OS (HR = 1.867, 95%CI: 1.341~2.598) and PFS (HR = 1.576, 95%CI: 1.146~2.167) compared to nCT+dCRT, further supporting the importance of surgery after tumor downstaging.

Neoadjuvant chemoradiotherapy combined with surgery is a recommended treatment for locally advanced resectable ESCC, which has been widely applied in clinical practice. However, there remains considerable debate over whether it is suitable for T4 stage ESCC patients (15, 16). Additionally, there is currently no direct evidence favoring one neoadjuvant treatment modalities, nCRT versus nCT, in terms of improving prognosis of ESCC patients. A study in 1997 reported that T4 stage ESCC patients who underwent surgery after nCT achieved a 5-year overall survival of 20% (with a median survival of 16 months), which increased to 29% (median survival of 23 months) in patients with R0 resection, findings that are consistent with our results. Csontos et al. (17) conducted a meta-analysis comparing nCRT and nCT for esophageal cancer, revealing that nCRT showed significant advantages only in pathological complete response rate (*P* < 0.001) and 30-day mortality rate (*P* = 0.015), with no significant differences in overall survival, local recurrence, distant metastasis, or other clinical/surgical complications. Wang et al. (18) conducted a prospective study on nCRT and nCT in ESCC patients, confirming that the former yielded a better pathological response, but there was no significant difference in recent OS. Therefore, it is still unclear which treatment protocol, nCRT or nCT, is more suitable for ESCC, and the choice of neoadjuvant treatment should be individualized based on the patient’s condition.

Notably, nCT efficacy critically guides clinical decision-making and correlates closely with patients’ long-term prognosis.For locally advanced tumors, favorable nCT response optimizes surgical conditions, boosting R0 resection rates and reducing short- and long-term recurrence;poor response necessitates timely regimen adjustment.Our follow-up data showed significantly longer DFS and overall survival OS in nCT-responsive versus non-responsive patients, consistent with domestic and international evidence, confirming nCT efficacy as a key prognostic predictor. Statistical analysis verified balanced nCT responses between experimental and control groups, with no significant differences in ORR, DCR, orCR/PR proportions (all P>0.05).This confirms comparable baseline characteristics, ensuring subsequent intergroup differences in efficacy and prognosis derive from interventions rather than nCT response discrepancy, thus guaranteeing study reliability. Moreover, subgroup conclusions after Bonferroni multiple testing correction further verified that nCT+S brought significant survival benefits to T4 ESCC patients with ECOG 0-1, cT4a, cN0, or mid-esophageal lesions, which provides more reliable evidence for clinical individualized treatment.

T4 ESCC is highly aggressive with poor natural prognosis, where R0 resection and R1 resection hold critical prognostic value and clinical significance. Clinical evidence confirms R0 resection significantly improves long-term survival, prolongs DFS and OS, and reduces local recurrence and distant metastasis by maximizing lesion clearance, reducing tumor burden and laying a foundation for adjuvant therapy. Given that T4 ESCC frequently invades adjacent vital organs, nCT efficacy is pivotal to achieving R0 resection, as effective nCT can downstage tumors, narrow invasion range and convert unresectable or positive-margin cases to R0-resectable ones. In contrast, R1 resection is an adverse prognostic factor for T4 ESCC, associated with higher local recurrence risk and poorer long-term survival than R0 resection. Pathologically confirmed R1 resection requires individualized adjuvant therapy based on general condition, tumor differentiation, lymph node metastasis and molecular characteristics to address residual lesions and reduce recurrence, yet the efficacy remains controversial. For T4 ESCC unresectable after nCT, blind radical surgery is inappropriate; palliative therapy should be administered promptly to improve quality of life, alleviate symptoms and avoid surgical trauma/complications worsening prognosis. Collectively, nCT efficacy is a key determinant of R0 resection feasibility and long-term prognosis in T4 ESCC; individualized surgical and adjuvant strategies based on nCT response are essential to maximize clinical benefits.

The significant advantage in OS and PFS observed with the nCT+S combined with postoperative adjuvant chemoradiotherapy treatment mode may be attributed to several factors. Neoadjuvant chemotherapy reduces tumor volume and downstage the tumor, thus improving the success rate and thoroughness of surgical resection (19). Surgry then directly remove tumor tissue and any surrounding potential metastatic lymph nodes, fundamentally reducing the tumor cell load and the risk of recurrence and metastasis. Furthermore, postoperative adjuvant therapy further eliminates subclinical lesions, reducing the chance of treatment failure after surgery (20, 21). In contrast, although chemoradiotherapy can kill tumor cells, ESCC is relatively insensitive to radiation, and radiotherapy may not fully eliminate tumor cells, increasing the risk of tumor residue and recurrence. Additionally, the adverse effects of chemoradiotherapy may impair the patient’s physical condition and limit their ability to tolerate subsequent treatments, negatively influencing survival.

Pre-treatment N stage was confirmed as an independent prognostic factor for ESCC patients In our study,. Univariate analysis showed that N stage was significantly correlated with OS and PFS, and multivariate analysis maintained its independence. Further subgroup analysis revealed that patients with N0 stage benefited more from the nCT+S treatment modality than those with N1 and N2 stage ESCC. This finding corroborates previous research indicating that the extent of lymph node metastasis is a critical determinant of patient outcomes (22–24). Patients with a higher N stage usually present with larger tumor burden, limited response to nCT, and poorer prognosis. A lower N stage often generally signifies limited local invasion and metastatic spread, which is associated with improved outcomes. In our study, N stage was independently associated with OS (HR = 1.595, 95%CI: 1.286~1.977) and PFS (HR = 1.741, 95%CI: 1.412~2.147), consistent with the long-term follow-up results of the CROSS trial (8), which also emphasized the importance of lymph node metastasis as an independent prognostic factor in esophageal cancer. Regardless of treatment modality, N stage holds pivotal value for prognostic stratification. In ESCC patients (25), N0 patients receiving nCT (DCF regimen) had significantly higher 3-year OS and PFS compared to N1/N2 patients, indicating the impact of the degree of lymph node metastasis on chemotherapy sensitivity. The NCCN guidelines (26) suggest that the recommended intensity of neoadjuvant treatment may vary according to lymph node status, with N0 patients being more likely to achieve R0 resection through chemotherapy, thereby enhancing prognosis. Therefore, N stage not only serves as a vital component of pre-treatment assessment but also offers important reference for post-treatment follow-up and individualized therapeutic strategies. For N0 patients, nCT may help reduce the risk of tumor recurrence and facilitate more complete surgical resections. For N1 and higher stage patients, more aggressive treatment strategies may be required, such as increasing chemotherapy dosage and duration or considering other treatments like radiotherapy or targeted therapies. While current research has confirmed the importance of N stage in assessing the prognosis of ESCC after nCT, some limitations exist. First, the accuracy of N stage is influenced by several factors, such as the sensitivity and specificity of imaging and the quality of pathological examinations. Second, the specific mechanisms by which N stage affects prognosis are not yet fully understood, necessitating further basic research. Future studies could focus on developing more accurate N stage assessment methods improve the accuracy of preoperative staging, investigating the molecular mechanisms of N stage’s impact on prognosis to identify new treatment targets, and conducting multicenter, large-sample clinical trials to further validate the prognostic value of N stage in different treatment protocols and patient populations.

Compared to nCT+dCRT, the nCT+S group had a lower rate of severe complications such as esophageal/anastomotic leaks and hematemesis. The higher incidence of esophageal leaks in T4 ESCC patients receiving radical chemoradiotherapy may be due to the more severe tumor infiltration and destruction of the esophageal wall, the cumulative cytotoxic effects of combined chemoradiotherapy, and the consequent impairment in tissue healing. Zhang et al. (11) reported an esophageal leak rate of 6.4% and a hematemesis rate of 2.1% in T4 ESCC patients and previous studies on T4 ESCC patients receiving dCRT documented leak rates ranging from 9% to 18% (27–29). These findings underscore the need for personalized dCRT regimens that take into account patient age, nutritional status, comorbidities, and the degree of tumor invasion to minimize the risk of such complications. Furthermore, although our study found no significant statistical difference in the overall treatment failure rate between the nCT+S and nCT+dCRT groups, the nCT+dCRT group experienced a significantly higher incidence of local tumor or regional lymph node recurrence compared to the nCT+S group. The elevated locoregional recurrence in T4 ESCC patients receiving dCRT likely results from several interrelated factors. First, the aggressive biology of T4 tumors, with their tendency to invade adjacent structures and spread via regional lymphatics, means that even after chemoradiotherapy, residual malignant cells may remain in the peritumoral tissue, seeding recurrence. Second, the inherent heterogeneity of ESCC means that subpopulations of tumor cells exhibit variable sensitivity to treatment, allowing resistant clones to persist and drive relapse. Third, to protect surrounding healthy tissues, radiotherapy may deliver suboptimal doses, and the extensive invasion seen in T4 tumors often complicates accurate delineation of the target area, leading to potential geographic misses. Finally, prolonged disease or previous treatments can contribute to the development of chemotherapy resistance, further undermining treatment efficacy and increasing the risk of local recurrence.

This study also has limitations that require further verification in future research. First, the sample size was relatively small, which may limit the statistical power and fail to fully reflect the efficacy differences between the two treatment modalities in a larger population. Second, the study participants were mainly from a single center, and the geographic and ethnic distribution of the patients may limit the generalizability and applicability of the findings. A major concern raised is potential selection bias because patients who became resectable after nCT were more likely to receive surgery, while unresectable cT4b patients tended to receive dCRT.We fully addressed this bias in two ways:①PSM balanced all baseline characteristics, including cT stage, tumor location, length, ECOG, and nCT response (all SMD < 0.2);②We further performed a prespecified subgroup analysis limited only to MDT-confirmed resectable T4 patients after nCT.Even in this homogeneous resectable subgroup, nCT+S remained significantly superior to nCT+dCRT.Therefore, the survival benefit is not an artifact of resectability selection, but reflects a real therapeutic advantage of surgery.Additionally, as a retrospective study, potential confounding factors, such as comorbidities, nutritional status, dietary habits, and quality of life, were not comprehensively accounted for.

For patients with cT4N0-3M0 ESCC who achieve effective response to neoadjuvant chemotherapy, the nCT+S strategy yields significantly better OS and PFS compared with nCT+dCRT, even when analysis is limited to MDT-confirmed resectable T4 cases.Subgroup analysis indicates that patients with ECOG 0-1, cT4a, cN0 stage, or mid-esophageal tumors may derive greater survival benefits from nCT+S.Additionally, nCT+S is associated with a lower incidence of severe treatment-related complications and a reduced risk of isolated local or regional lymph node recurrence.Given the limitations of this single-center retrospective study, further large-scale, multicenter randomized controlled trials are warranted to validate these findings and establish the optimal standard treatment regimen for T4 ESCC patients.

## Data Availability

The raw data supporting the conclusions of this article will be made available by the authors, without undue reservation.

## References

[1] Morgan E Soerjomataram I Rumgay H Coleman HG Thrift AP Vignat J . The global landscape of esophageal squamous cell carcinoma and esophageal adenocarcinoma incidence and mortality in 2020 and projections to 2040: New estimates from GLOBOCAN 2020. Gastroenterology. (2022) 163:649–658.e2. doi: 10.1053/j.gastro.2022.05.054. PMID: 35671803

[2] Zhu H Ma X Ye T Wang H Wang Z Liu Q . Esophageal cancer in China: Practice and research in the new era. Int J Cancer. (2023) 152:1741–51. doi: 10.1002/ijc.34301. PMID: 36151861

[3] Deboever N Jones CM Yamashita K Ajani JA Hofstetter WL . Advances in diagnosis and management of cancer of the esophagus. BMJ. (2024) 385:e074962. doi: 10.1136/bmj-2023-074962. PMID: 38830686

[4] Akutsu Y Matsubara H . Chemoradiotherapy and surgery for T4 esophageal cancer in Japan. Surg Today. (2015) 45:1360–5. doi: 10.1007/s00595-015-1116-4. PMID: 25583206

[5] Qi C Hu L Zhang C Wang K Qiu B Yi J . Role of surgery in T4N0-3M0 esophageal cancer. World J Surg Oncol. (2023) 21:369. doi: 10.1186/s12957-023-03239-8. PMID: 38008742 PMC10680323

[6] Yang W Niu Y Sun Y . Current neoadjuvant therapy for operable locally advanced esophageal cancer. Med Oncol. (2023) 40:252. doi: 10.1007/s12032-023-02097-4. PMID: 37498350

[7] Yang H Liu H Chen Y Zhu C Fang W Yu Z . Long-term efficacy of neoadjuvant chemoradiotherapy plus surgery for the treatment of locally advanced esophageal squamous cell carcinoma: The NEOCRTEC5010 randomized clinical trial. JAMA Surg. (2021) 156:721–9. doi: 10.1001/jamasurg.2021.2373. PMID: 34160577 PMC8223138

[8] Eyck BM van Lanschot JJB Hulshof MCCM van der Wilk BJ Shapiro J van Hagen P . Ten-year outcome of neoadjuvant chemoradiotherapy plus surgery for esophageal cancer: The randomized controlled CROSS trial. J Clin Oncol. (2021) 39:1995–2004. doi: 10.1200/JCO.20.03614. PMID: 33891478

[9] Zeng H Zhang F Sun Y Li S Zhang W . Treatment options for neoadjuvant strategies of esophageal squamous cell carcinoma (Review). Mol Clin Oncol. (2023) 20:4. doi: 10.3892/mco.2023.2702. PMID: 38223404 PMC10784769

[10] Gao LR Li C Han W Ni W Deng W Tan L . Survival benefit of surgery in patients with clinical T4 esophageal cancer who achieved complete or partial response after neoadjuvant chemoradiotherapy or radiotherapy. Ther Adv Med Oncol. (2022) 14:17588359221108693. doi: 10.1177/17588359221108693. PMID: 35923925 PMC9340417

[11] Zhang T Guo Z Chen X Dong J Jiang H Tang P . A retrospective study comparing definitive chemoradiotherapy vs. chemoradiotherapy followed by surgery in T4 esophageal squamous cell carcinoma patients who were downstaged after neochemoradiotherapy. Radiat Oncol. (2022) 17:148. doi: 10.1186/s13014-022-02116-0. PMID: 35999608 PMC9396773

[12] Allum WH Stenning SP Bancewicz J Clark PI Langley RE . Long-term results of a randomized trial of surgery with or without preoperative chemotherapy in esophageal cancer. J Clin Oncol. (2009) 27:5062–7. doi: 10.1200/JCO.2009.22.2083. PMID: 19770374

[13] Cunningham D Allum WH Stenning SP Thompson JN Van de Velde CJ Nicolson M . Perioperative chemotherapy versus surgery alone for resectable gastroesophageal cancer. N Engl J Med. (2006) 355:11–20. doi: 10.1056/NEJMoa055531. PMID: 16822992

[14] Ando N Kato H Igaki H Shinoda M Ozawa S Shimizu H . A randomized trial comparing postoperative adjuvant chemotherapy with cisplatin and 5-fluorouracil versus preoperative chemotherapy for localized advanced squamous cell carcinoma of the thoracic esophagus (JCOG9907). Ann Surg Oncol. (2012) 19:68–74. doi: 10.1245/s10434-011-2049-9. PMID: 21879261

[15] Sugawara K Yagi K Okumura Y Nishida M Aikou S Yamashita H . Long-term outcomes of multimodal therapy combining definitive chemoradiotherapy and salvage surgery for T4 esophageal squamous cell carcinoma. Int J Clin Oncol. (2020) 25:552–60. doi: 10.1007/s10147-019-01590-z. PMID: 31828451

[16] Shiraishi O Yasuda T Kato H Momose K Hiraki Y Yasuda A . Comparison of aggressive planned salvage surgery versus neoadjuvant chemoradiotherapy plus surgery for borderline resectable T4 squamous cell carcinoma. Ann Surg Oncol. (2021) 28:6366–75. doi: 10.1245/s10434-021-09875-2. PMID: 33768398

[17] Csontos A Fazekas A Szakó L Farkas N Papp C Ferenczi S . Effects of neoadjuvant chemotherapy vs chemoradiotherapy in the treatment of esophageal adenocarcinoma: A systematic review and meta-analysis. World J Gastroenterol. (2024) 30:1621–35. doi: 10.3748/wjg.v30.i11.1621. PMID: 38617451 PMC11008422

[18] Wang H Tang H Fang Y Tan L Yin J Shen Y . Morbidity and mortality of patients who underwent minimally invasive esophagectomy after neoadjuvant chemoradiotherapy vs neoadjuvant chemotherapy for locally advanced esophageal squamous cell carcinoma: A randomized clinical trial. JAMA Surg. (2021) 156:444–51. doi: 10.1001/jamasurg.2021.0133. PMID: 33729467 PMC7970392

[19] Altorki N Harrison S . What is the role of neoadjuvant chemotherapy, radiation, and adjuvant treatment in resectable esophageal cancer. Ann Cardiothorac Surg. (2017) 6:167–74. doi: 10.21037/acs.2017.03.16. PMID: 28447006 PMC5387137

[20] An Q Su Y Wang Y Zhen C Bai W Fu L . Is postoperative adjuvant radiotherapy necessary for patients with esophageal cancer after neoadjuvant chemoradiotherapy? An analysis based on the SEER database. Saudi Med J. (2024) 45:900–10. doi: 10.15537/smj.2024.45.9.20240045. PMID: 39218457 PMC11376696

[21] Zeng Y Su X Zhou T Jia J Liu J Yu W . Propensity-matched study on locally advanced esophageal cancer: Surgery versus post-operative radiotherapy. Radiat Oncol. (2024) 19:130. doi: 10.1186/s13014-024-02528-0. PMID: 39334405 PMC11428459

[22] Hamai Y Emi M Ibuki Y Kurokawa T Yoshikawa T Ohsawa M . Distribution of lymph node metastasis in esophageal squamous cell carcinoma after trimodal therapy. Ann Surg Oncol. (2021) 28:1798–807. doi: 10.1245/s10434-020-09106-0. PMID: 32885399

[23] Leng X He W Yang H Chen Y Zhu C Fang W . Prognostic impact of postoperative lymph node metastases after neoadjuvant chemoradiotherapy for locally advanced squamous cell carcinoma of esophagus: From the results of NEOCRTEC5010, a randomized multicenter study. Ann Surg. (2021) 274:e1022–9. doi: 10.1097/SLA.0000000000003727. PMID: 31855875

[24] Yeung JC Bains MS Barbetta A Nobel T DeMeester SR Louie BE . How many nodes need to be removed to make esophagectomy an adequate cancer operation, and does the number change when a patient has chemoradiotherapy before surgery. Ann Surg Oncol. (2020) 27:1227–32. doi: 10.1245/s10434-019-07870-2. PMID: 31605332 PMC7561013

[25] Kato K Machida R Ito Y Daiko H Ozawa S Ogata T . Doublet chemotherapy, triplet chemotherapy, or doublet chemotherapy combined with radiotherapy as neoadjuvant treatment for locally advanced oesophageal cancer (JCOG1109 NExT): A randomised, controlled, open-label, phase 3 trial. Lancet. (2024) 404:55–66. doi: 10.1016/S0140-6736(24)00745-1. PMID: 38876133

[26] Ajani JA D'Amico TA Bentrem DJ Cooke D Corvera C Das P . Esophageal and esophagogastric junction cancers, version 2.2023, NCCN clinical practice guidelines in oncology. J Natl Compr Canc Netw. (2023) 21:393–422. doi: 10.6004/jnccn.2023.0019. PMID: 37015332

[27] Nishimura Y Suzuki M Nakamatsu K Kanamori S Yagyu Y Shigeoka H . Prospective trial of concurrent chemoradiotherapy with protracted infusion of 5-fluorouracil and cisplatin for T4 esophageal cancer with or without fistula. Int J Radiat Oncol Biol Phys. (2002) 53:134–9. doi: 10.1016/s0360-3016(01)02813-9. PMID: 12007951

[28] Ohtsu A Boku N Muro K Chin K Muto M Yoshida S . Definitive chemoradiotherapy for T4 and/or M1 lymph node squamous cell carcinoma of the esophagus. J Clin Oncol. (1999) 17:2915–21. doi: 10.1200/JCO.1999.17.9.2915. PMID: 10561371

[29] Kaneko K Ito H Konishi K Kurahashi T Ito T Katagiri A . Definitive chemoradiotherapy for patients with Malignant stricture due to T3 or T4 squamous cell carcinoma of the oesophagus. Br J Cancer. (2003) 88:18–24. doi: 10.1038/sj.bjc.6600684. PMID: 12556953 PMC2376792

